# Dendritic Cells Exposed to MVA-Based HIV-1 Vaccine Induce Highly Functional HIV-1-Specific CD8^+^ T Cell Responses in HIV-1-Infected Individuals

**DOI:** 10.1371/journal.pone.0019644

**Published:** 2011-05-18

**Authors:** Núria Climent, Susana Guerra, Felipe García, Cristina Rovira, Laia Miralles, Carmen Elena Gómez, Núria Piqué, Cristina Gil, José María Gatell, Mariano Esteban, Teresa Gallart

**Affiliations:** 1 Service of Immunology, Hospital Clínic de Barcelona, Barcelona, Spain; 2 Institut d'Investigacions Biomèdiques August Pi i Sunyer (IDIBAPS)-AIDS Research Group, and Catalonian Center for HIV Vaccines (HIVACAT), Barcelona, Spain; 3 Department of Molecular and Cellular Biology, Centro Nacional de Biotecnología CSIC, Campus Universidad Autónoma, Madrid, Spain; 4 Department of Preventive Medicine and Public Health, Universidad Autónoma, Madrid, Spain; 5 Infectious Diseases and AIDS Unit, Hospital Clínic de Barcelona, Barcelona, Spain; 6 Department of Microbiology and Parasitology, Pharmacy Faculty, Universitat de Barcelona, Spain; University of Palermo, Italy

## Abstract

Currently, MVA virus vectors carrying HIV-1 genes are being developed as HIV-1/AIDS prophylactic/therapeutic vaccines. Nevertheless, little is known about the impact of these vectors on human dendritic cells (DC) and their capacity to present HIV-1 antigens to human HIV-specific T cells. This study aimed to characterize the interaction of MVA and MVA expressing the HIV-1 genes Env-Gag-Pol-Nef of clade B (referred to as MVA-B) in human monocyte-derived dendritic cells (MDDC) and the subsequent processes of HIV-1 antigen presentation and activation of memory HIV-1-specific T lymphocytes. For these purposes, we performed *ex vivo* assays with MDDC and autologous lymphocytes from asymptomatic HIV-infected patients. Infection of MDDC with MVA-B or MVA, at the optimal dose of 0.3 PFU/MDDC, induced by itself a moderate degree of maturation of MDDC, involving secretion of cytokines and chemokines (IL1-ra, IL-7, TNF-α, IL-6, IL-12, IL-15, IL-8, MCP-1, MIP-1α, MIP-1β, RANTES, IP-10, MIG, and IFN-α). MDDC infected with MVA or MVA-B and following a period of 48 h or 72 h of maturation were able to migrate toward CCL19 or CCL21 chemokine gradients. MVA-B infection induced apoptosis of the infected cells and the resulting apoptotic bodies were engulfed by the uninfected MDDC, which cross-presented HIV-1 antigens to autologous CD8^+^ T lymphocytes. MVA-B-infected MDDC co-cultured with autologous T lymphocytes induced a highly functional HIV-specific CD8^+^ T cell response including proliferation, secretion of IFN-γ, IL-2, TNF-α, MIP-1β, MIP-1α, RANTES and IL-6, and strong cytotoxic activity against autologous HIV-1-infected CD4^+^ T lymphocytes. These results evidence the adjuvant role of the vector itself (MVA) and support the clinical development of prophylactic and therapeutic anti-HIV vaccines based on MVA-B.

## Introduction

Since its sudden emergence in 1981, the HIV/AIDS infection, which spread rapidly worldwide, has resulted in more than 60 million of infected people and more than 25 million deaths, and remains a priority in the global public health as it continues to expand. Indeed, currently there are more than 33 million of infected individuals, with an incidence per year of around 3 million of newly infected persons and 2 million deaths (www.unaids.org). Although since 1996 very effective antiretroviral medications have been developed, which allow the control, in most patients, of HIV-1 replication [1], this therapy is not a cure as it fails to eradicate the virus and must be maintained for life, involving the risks of the appearance of drug resistances and adverse effects. In addition, given the high economic cost of this therapy and the high degree of sanitary requirements that involves, it is not universally accessible in resource-limited areas of the world, where most of infected persons live. Therefore, the development of a safe and effective preventive HIV-1 vaccine is a global public-health priority to try to halt the HIV-1 pandemic [2]–[5].

Initial efforts in the development of an HIV-1 vaccine were aimed at generating neutralizing antibodies (nAb) against the envelope (Env) protein using recombinant gp120. Two of these Env-based vaccines formulated in conventional adjuvant (alum) were tested in phase III clinical trials without any protective effect [6], [7]. The great difficulty in inducing nAb and the growing body of evidence demonstrating the important role of cytotoxic T lymphocytes (CTL) to control HIV-1 infection prompted the interest to develop T cell-based vaccines [2], [3], [5], [8], [9]. These T-cell vaccines have been developed using HIV-1 plasmid DNA and HIV-1 genes inserted in viral vectors, mainly adenovirus vectors and poxvirus vectors. From studies in non human primates (NHP), T-cell vaccines are unlikely to impede the infection but rather can help to control HIV-1 replication after infection, reducing the rate of disease progression. A notable number of these type of vaccines have been clinically tested but only four of them have reached efficacy trials (phases III or IIb). The results of two of these efficacy trials (STEP and Phambili trials) employing adenovirus serotype 5 (Ad5)-based vaccine with inserted gag-pol-nef genes of HIV-1 clade B, were deeply disappointing, as the vaccination not only failed to reduce the viral load after HIV-1 infection, but the incidence of HIV-1 infection in the STEP trial was higher among vaccinated individuals than in placebo-treated subjects [10]–[12]. Nevertheless, promising results were obtained in the more recent efficacy trial with 16,402 volunteers in Thailand, using a prime-boosting vaccination with a canarypox virus vectoring Env-Gag-Pol HIV-1 genes (ALVAC-HIV) and a trimeric recombinant gp120 (AIDSVAX B/E).Compared with placebo, 31% of vaccinees were protected against HIV-1 infection [13]. Although very modest, this rate of efficacy is relevant because is the first description indicating that a protective immunization against HIV-1 might be feasible. This study also underscores the relevance of characterizing other poxvirus, such as MVA, as vaccine candidates [14]–[16].

As anti-HIV vaccine candidates, attenuated poxvirus vectors have a number of desirable features, including promising safety profiles, the ability to incorporate substantial genetic material for the expression of foreign gene products and the capacity to efficiently activate the host immune responses to virally expressed antigens [16]–[19].

Among poxviruses, MVA vectors (the attenuated poxvirus strain modified vaccinia virus Ankara) have already generated very promising results in *in vitro* studies [17], [20] and in simian model of HIV infection [21], [22] and, on this basis, they are also currently undergoing evaluation in human trials [23]–[26]. MVA expressing Env-Gag-Pol-Nef HIV genes of clade B (MVA-B) has been developed to pharmaceutical GMP degree and it is currently being tested as either preventive vaccination in clinical trials in Spain and it is expected to be tested as therapeutic vaccine in the near future.

Moreover, previous non clinical studies have evaluated the interaction of these vectors (MVA and MVA expressing HIV genes) with myeloid derived dendritic cells (MDDC) using both *ex vivo* [17], [18], [20], [27] and animal models [28], showing that combination of MDDC with these MVA vectors are able to enhance cellular responses against HIV [17], [20].

It is known the ability of dendritic cells (DC) to induce strong cell-mediated immune responses by promoting the effector functions of T helper (Th)-1 cells, cytotoxic T lymphocytes (CTL) and natural killer (NK) cells, positioning them at the fore-front of therapeutic applications in HIV infection [29]–[31] and also in cancer treatment [32], [33]. Results of studies in mouse models suggest that immunization with DC loaded with HIV-1 viral lysates, envelope glycoproteins or inactivated virus mount a potent immune response against HIV [29], [34], [35]. In this context, the objective of the present study was to study the interaction of MVA-B with MDDC and the capacity of these cells to process and present the HIV-1 antigens to autologous human T cells from HIV-1-infected patients, in a 6–7 day co-culture system. To know how MVA and MVA-B interact with MDDC is of paramount importance for an adequate design of an efficient anti-HIV vaccine based on MVA-B to be tested at both non clinical and clinical studies. Results of the present study have demonstrated that interaction of MVA-B with MDDC from HIV-infected patients induces a strong HIV-specific CD8^+^ T cell response, thus supporting the clinical development of a prophylactic and therapeutic anti-HIV vaccine based on MVA-B.

## Materials and Methods

### Ethics Statement

This study received the approval of the Committee of Ethics and Clinical Investigation of the Hospital Clínic Universitari (Barcelona, Spain). All the subjects participating in the study were recruited at the Service of Infectious Diseases & AIDS Unit of this Hospital and gave their informed written consent.

### Study individuals

Samples of EDTA-anticoagulated venous blood samples were obtained from asymptomatic HIV-1-infected patients with baseline CD4^+^ T cell counts >450 cells/mm^3^, and plasma viral load (pVL) <10,000 HIV-1 RNA copies/ml.

### Generation of MDDC

Peripheral blood mononuclear cells (PBMC) were isolated immediately after venous extraction by using a standard Ficoll gradient. Cells were processed immediately after isolation. To obtain human monocytes, PBMC were incubated in plastic plates (2 h at 37°C) in a humidified atmosphere with 5% CO_2_ in MDDC medium (serum-free XVIVO-15 medium, Lonza, Maryland, USA) supplemented with 1% autologous serum, gentamicin (B/Braun Medical, Melsungen, Germany) and fungizone (amphotericin B, Bristol-MyersSquibb, Rueil-Malmaison, France) and 1 µM zidovudine (Retrovir from GlaxoSmithKline, Madrid, Spain) to avoid HIV infection. To obtain immature MDDC, adherent cells were washed four times with pre-warmed MDDC medium and then cultured for 5 days in the presence of 1000 U/ml each of recombinant human IL-4 (Strathmann Biotec AG, Hamburg, Germany) and recombinant human GM-CSF (Peprotech, London, UK) on days 0 and 2. The purity and immunophenotype were assessed by flow cytometry (see below).

### Virus

The poxvirus strains used in this work included modified vaccinia virus Ankara (MVA) obtained after 586 passages in CEF cells (derived from clone F6 at passage 585) and the recombinant MVA expressing the HIVBX08 gp120 and HIVIIIB Gag-Pol-Nef genes from clade B (MVA-B). This nucleotide polygene construct encodes a scrambled Nef variant lacking biological activity [28], [36]. Viruses were grown in primary CEF cells, purified through two sucrose cushions and titrated by immunostaining using rabbit polyclonal anti-vaccinia virus serum as previously described [37].

### MDDC infection with MVA and MVA-B viruses and MDDC maturation

Immature MDDC in MDDC medium were exposed to MVA and MVA-B vectors at a multiplicity of infection (MOI) of 0.003 to 10 PFU/MDDC. One hour later, fresh MDDC medium, IL-4 and GM-CSF (1000 U/ml each) were added. To obtain mature MDDC, a cocktail of recombinant human cytokines containing TNF-α, IL-6 (1000 IU/ml each, Strathmann Biotec AG), IL-1β (300 UI/ml, Strathmann Biotec AG) and PGE_2_ (1 µg/ml, Pfizer, Madrid, Spain) was added at 2 h post-infection, and the mixture was incubated for a whole period of 48 h. In parallel, for some experiments, MDDC were pulsed with recombinant soluble p24 (Protein Sciences, Meriden, CT) at 1 µg/ml. Samples from each experiment were set aside to evaluate intracellular expression of Gag protein, viral protein expression using polyclonal antibodies against the WR strain of vaccinia virus, the expression of the cell-surface markers (at 16 h post-infection) and cytokine secretion (at 16 h and 48 h post-infection). The purity and immunophenotype of mature MDDC were assessed by flow cytometry analysis using commercially labeled monoclonal antibodies (mAbs) against surface markers (see below, “flow cytometry”).

### Viability evaluation of MVA- or MVA-B-infected MDDC

The dual staining method with annexin V and 7-amino-actinomycin (7AAD) was used, which allows to separate live cells as negative for both dyes, dead cells as positive for 7AAD or for both markers and apoptotic cells as positive for annexin V but negative for 7AAD. Approximately 2×10^5^ pooled cells in 100 µl of MDDC medium were added to individual wells in a round-bottomed microtiter plate. Cells were washed with FACS buffer and resuspended in 100 µl of annexin V binding buffer and then FITC-annexin V was added (5 µg) to each well according to the manufacturer's protocol (ApoDETECT FITC-Annexin V Kit; Zymed Laboratories, San Francisco, CA, USA). After 15 min of incubation, 1 µg of 7AAD (Molecular Probes, Eugene, OR, USA) was added to each well and the cells were incubated for 15 min before flow cytometry analysis in a FACSalibur flow cytometer ABC (BD Biosciences, San Diego, CA, USA). These experiments were done in triplicates.

### Flow cytometry

The immunophenotype of immature and mature MDDC was assessed by two-colour flow cytometry using FITC- and PE-conjugated mAbs against CD14, CD25, CD80, CD83, CD86, CD11c, CD40 and HLA-DR and HLA-ABC (BD Biosciences). Also, CD3, CD19 and CD56 were used to assess the purity of the cell cultures, resulting to be lower than 2% of the total population. FITC- and PE-conjugated isotype-matched monoclonal antibodies of irrelevant specificity were used as negative controls. Intracellular staining of Gag protein was performed as described elsewhere [38], [39] with minor modifications. Briefly, after staining MDDC populations for cell-surface PE-CD86, cell were fixed and permeabilized with BD Cytofix/Cytoperm kit (BD Biosciences) and then incubated with anti-HIV Gag antibody conjugated to fluorescein isothiacyanate (clone KC57; Beckman Coulter, Fullerton, CA, USA) in Perm/Wash buffer. The cells were analyzed in a FACScan flow cytometer (BD Biosciences). Data obtained were analyzed with the FlowJo software (Tree Star, Inc. Ashland, OR, USA). Expression of surface and intracellular markers was measured by the percentage of positive cells and the geometric mean fluorescence intensity. Cell populations were selected by forward and side light-scatter parameters.

### Fluorescence microscopy

Intracellular staining of Gag protein was performed as described above. Intracellular staining of WR protein was performed as described before with minor modifications [18]. Briefly, MDDC populations were washed with PBS, fixed with 4% paraformaldehyde and permeabilized with BD Cytofix/Cytoperm kit. Cells were incubated with primary anti-WR (anti-VV, obtained from R. Blasco, INIA, Spain) antibodies, followed by FITC secondary antibodies (BD Biosciences). Phagocytosis was further confirmed by fluorescence microscopy. After the co-culture of uninfected MDDC with CFSE-labelled MVA-B-infected MDDC (see below) during 48 h with maturation cocktail, the cells were washed with PBS and stained with PE-anti-CD80 antibody (BD Biosciences) at 4°C. In all the experiments, nucleus was stained for 10 minutes using Hoechst staining and the cells were adhered on a slide with Poly-L-Lysine (SIGMA- Aldrich, Madrid, Spain) for 30 minutes. After that, the cells were observed under Nikon Eclipse E600 fluorescence microscope for green, red and blue fluorescence.

### Cytokine and chemokine secretion by MDDC infected with MVA and MVA-B

The secretion of cytokines by MDDC infected with MVA or MVA-B was measured in the culture supernatant using the Luminex technology (Cytokine Human 25-Plex Planel, Invitrogen, Carlsbad, CA, USA), following the manufacturer's instructions. The following 25 mediators were tested: Eotaxin, GM-CSF, IL-1β, IL-1RA, IL-2, IL-2R, IL-4, IL-5, IL-6, IL-7, IL-8, IL-10, IL-12p40/p70, IL-13, IL-15, IL-17, IFN-α, IFN-γ, IP-10, MCP-1, MIG, MIP-1α, MIP-1β, RANTES and TNF-α.

### Activation of HIV-1-specific T lymphocytes by autologous MDDC infected with MVA-B

As a source of enriched T cells we employed fresh PBMC depleted of monocytes after adherence to plastic as indicated above for the generation of MDDC. These monocyte-depleted lymphocytes were washed (3×) and resuspended in serum-free XVIVO-10 medium and labeled with CFSE following the instructions of the manufacturer (CellTrace CFSE cell proliferation kit, Molecular Probes, Paisley, UK). Autologous infected and matured MDDC (6×10^4^ MDDC/well) were washed (4×) and resuspended in XVIVO-10 and co-cultured with autologous fresh CFSE-labeled lymphocytes (2×10^5^ T-lymphocytes/well) in a final volume of 0.2 ml in XVIVO-10 medium supplemented with 1 µM zidovudine to impede possible replication of endogenous HIV-1. Infection with MVA or MVA-B and pulsing with soluble recombinant HIV-1 p24, and maturation of MDDC were performed as indicated above. The contribution of MDDC alone and monocyte depleted PBMC (lymphocytes) alone was determined as negative controls of proliferation and cytokine secretion. The co-cultures were done in triplicates at 37°C in a humidified atmosphere of air with 5% CO_2_. After 6–7 days, proliferating CD4^+^ (CD3^+^CD8^−^) and CD8^+^ (CD3^+^CD8^+^) T cells were determined by direct staining with mAbs conjugated with α-CD3-Per-CP and α-CD8-PE. Mouse Ig isotypes mAbs (from BD Biosciences) of unknown antigen specificity conjugated with PerCP or PE were used as negative control mAbs. The stained cells were analyzed on a FACSCalibur flow cytometer (BD Biosciences). T cell populations were selected by forward and side light-scatter parameters and sub-gated for CD4 or CD8 expression. Cells that proliferated after the co-culture had lower intensity of CFSE (CFSE^low^) in comparison with basal conditions. MVA, MVA-B or p24 specific proliferation was expressed as the percentage of CFSE^low^ cells after co-culture with MDDC infected with MVA or MVA-B or pulsed with soluble p24 minus the percentage of (mock-treated) uninfected CFSE^low^ cells. Aside from the CFSE- proliferation of CD4^+^ and CD8^+^ T cells, the levels of IFN-γ, IL-2, IL-6 and TNF-α secreted in the co-culture supernatants were also quantified at days 2 and 6 using the CBA assay (BD Bioscience) and MIP-1α, MIP-1β, and RANTES were quantified using the Luminex technology (see above).

### Cytotoxicity assay

Effector cells were autologous monocyte-depleted PBMC (lymphocytes) co-cultured for 6 days with MDDC mock-treated or infected with MVA or MVA-B (MDDC∶lymphocytes ratio of 1∶10). After this co-culture period, CFSE staining of effector cells was performed one day before the cytotoxicity assay. Target cells were autologous CD8 and monocyte-depleted PBMC; depletion of CD8^+^ cells (98%) was done with CD8-coupled MACS microbead columns and MACS cell separator (Miltenyi Biotec, Inc). CD8-depleted T cells (70% CD4^+^ T cells) were activated with PHA in the presence of IL-2 (Proleukin, Chiron Corp, California, USA) for 72 h before infection with a clade B X4R5 tropic HIV-1 virus isolated from an HIV-infected patient (>200 pg of HIV-p24 at a MOI of 0.1). After two days of infection, and also one day before the cytotoxicity assay, determination of intracellular Gag expression by flow cytometry was performed in target cells to confirm fully HIV infection (93.17% of Gag-positive cells was and 42,26% of viable cells). Target cells were washed and seeded at 5×10^3^ cells/well at an effector/target ratio of 10∶1 for 4 hours in triplicates and then cells were collected and stained with anti-CD3-PerCP and anti-CD8-PE and Annexin V-FITC. Cells were resuspended in a final volume of 120 µl and were acquired during 60 seconds (at 1 µl/second). The cells were analyzed and counted by Flow cytometry. Number of live target cells was assessed based on SSC and FSC parameters (gate of live activated lymphocytes), and CFSE, Annexin V, CD3 and CD8 labelling. Live CD4^+^ cell count corresponded to the phenotype: CFSE-negative, CD3-positive and CD8-negative, Annexin V-negative.

### MDDC migration to a chemokine gradient of CCL19 or CCL21

The migration of MDDC toward CCL19 or CCL21 chemokines (from PeproTech, London, UK) was measured in a 96- plate transwell chemotaxis chambers (Corning Costar, Cambridge, MA, USA), using a polycarbonate filter of 5 µm pore size. Briefly, 150 µl of recombinant human CCL19 (300 ng/ml) or recombinant human CCL21 (250 ng/ml) or medium alone were placed in the lower wells. Upper wells were loaded with 50 µl of infected MDDC cell suspensions (infected with MVA and MVA-B at 0.3 PFU/MDDC or mock infected) and maturated as above described during 48 h and 72 h at a concentration of 10^6^ MDDC/ml in serum-free XVIVO-15 medium. Each condition was set up in triplicates. The complete chamber was kept in a humidified atmosphere with 5% CO_2_ at 37°C for 3 h. Thereafter, cell suspensions in the upper well were removed and cells that had migrated through the filter to the lower wells were counted by flow cytometry for 1 minute. Values were given as percentage of migrated cells ± SEM in relation to the initial added cells. Subsequently, migrated MDDC were collected and co-cultured with CFSE labeled autologous lymphocytes at a 1∶40 ratio (5×10^3^ MDDC: 2×10^5^ lymphocytes/well) in triplicates. Six days later, proliferation was tested by flow cytometry using the CFSE assay as described above.

### Phagocytosis assay

The phagocytosis assay was performed as reported elsewhere with minor modifications [40]. Immature MDDC were labeled with CFSE following the manufacturer's instructions and infected with MVA-B at a MOI of 10 PFU/MDDC for 1 h (infected DC, CFSE^+^). They were then extensively washed and mixed with non-labeled and uninfected immature MDDC at a 2∶1 ratio. One hour later, fresh MDDC medium containing IL-4 and GM-CSF was added. At 2 h post-infection, the maturation cocktail was added and the mixture was incubated for a whole period of 48 h. As negative controls, uninfected cells and infected cells were separately cultured at the same conditions. Wells containing cell mixtures and control cells were then washed several times with cold PBS to aspirate noningested MDDC microvesicles. All the cells were labeled with a PE-conjugated anti-CD80 mAb and analyzed by FACS. CD80-PE labeled exclusively uninfected MDDC because MVA-B-infected MDDC die following 48 post-infection and do not undergo sufficient maturation to achieve the expression of this marker as they die (see results). MDDC that ingested apoptotic bodies derived from MVA-infected MDDC were identified by their positive signal for both CFSE and CD80-PE. Phagocytosis was further confirmed by fluorescence microscopy.

### Analysis of HIV antigen cross-presentation by MVA-B-infected MDDC

To assess whether the mechanism of HIV-1 antigen presentation by MVA-B-infected MDDC to autologous CD8^+^ T cells was cross-presentation the following procedures were done. On day 2, the initial MDDC culture (at a dose of 6×10^4^ cells/well) was divided in two different cultures. On the one hand, one third of the initial cell load (2×10^4^ cells/well) was exposed to MVA-B at the highest tested concentration of 10 PFU/MDDC. One hour later, cells were extensively washed, fresh MDDC medium containing IL-4 and GM-CSF was added and the cells were incubated for 72 h (total culture time: 5 days). On the other hand, two thirds of the initial culture (4×10^4^ cells/well) were incubated for 3 additional days in MDDC medium containing IL-4 and GM-CSF (total culture time: 5 days) (uninfected immature MDDC). At this time, the whole content of infected MDDC, consisting of a mixture of apoptotic cells and apoptotic bodies, was incubated for two hours with the other two thirds of the initial culture (uninfected immature MDDC), and then the maturation cocktail containing TNF-α, IL-1β, IL-6 and PGE_2_ was added and the mixture was incubated for 48 h. Afterwards, this mixture of MDDC were co-cultured for 6–7 days with autologous T lymphocytes to assess T cell proliferation as above indicated. In some experiments, to further exclude a direct antigen presentation mechanism by infected MDDC, the one third of MVA-B-infected MDDC were heat-inactivated for 1 hour at 55°C.

### Statistical analysis

Data were analyzed using the GraphPad Prism software (GraphPad Software, San Diego, CA, USA). Comparisons among groups were performed by unpaired and paired Student's t-test. For the analysis of correlation the non-parametric test of Spearman was applied. For all tests used, two sided p values less than 0.05 were considered to indicate statistical significance. Correlation coefficients (r^2^) were used to evaluate the goodness of fit of linear and curve models.

## Results

### Upon MVA-B infection, MDDC expressed intracellular Gag protein in a dose-dependent manner

MDDC were generated from samples of chronically HIV-1-infected patients with baseline CD4^+^ T lymphocytes above 450 cells/mm^3^, and pVL below 10,000 HIV-1 RNA copies/ml.

**Figure 1:**
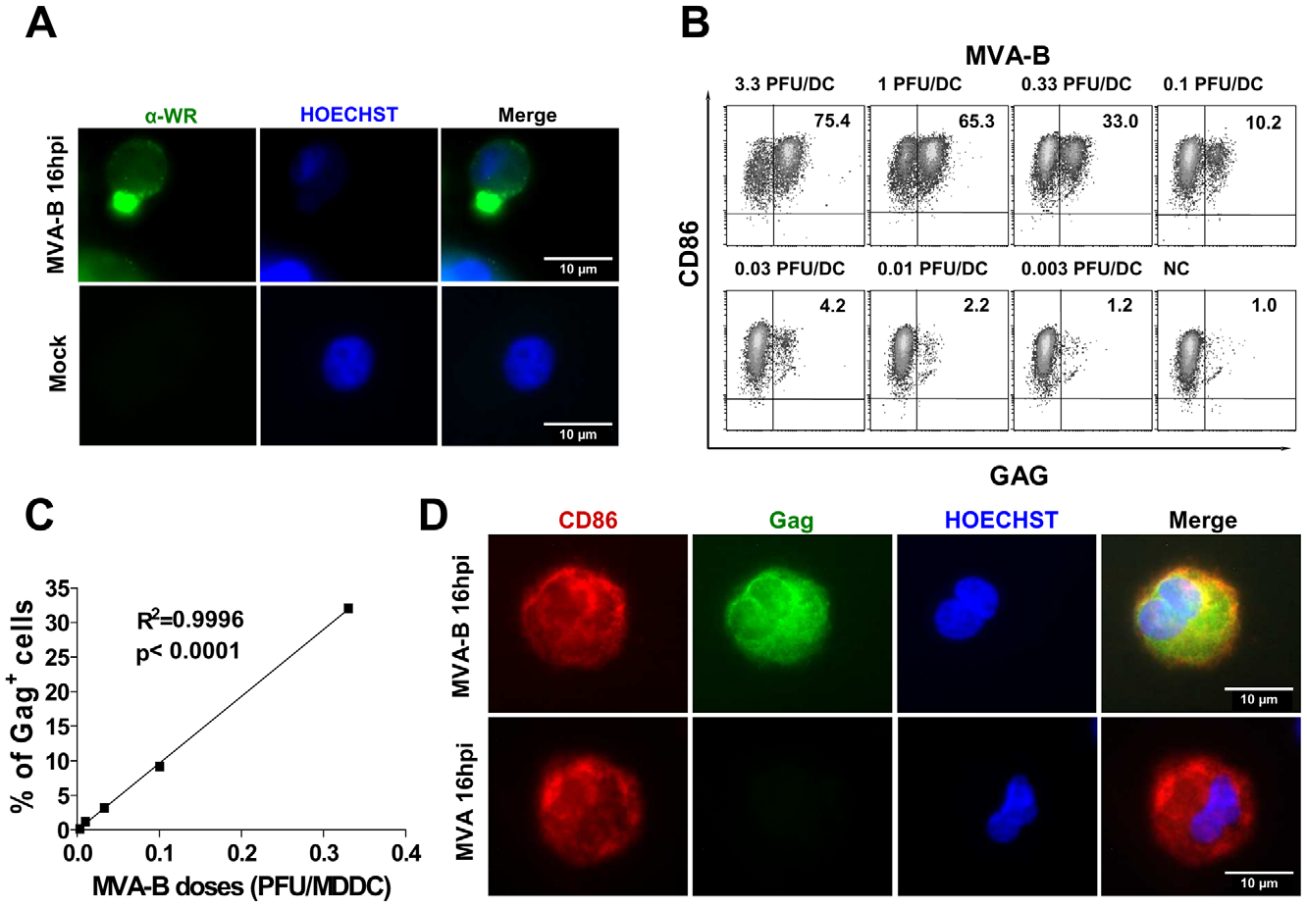
MDDC infected with MVA-B expressed intracellular Gag protein in a dose-dependent manner. MDDC were infected with serial three-fold dilutions of a stock of MVA-B, from 0.003 to 10 PFU/MDDC. After MVA-B infection of MDDC, Gag and CD86 expression was measured by flow cytometry. (A) Intracellular viral protein expression in MDDC observed by fluorescence microscopy. MDDC at 16 h post-infection not infected (Mock) or infected with MVA-B (at 10 PFU/MDDC) were fixed-permeabilized and viral proteins were stained indirectly with FITC (green) and nucleus was visualized using Hoechst (blue). These data are representative of two individual experiments. (B) Density plots showing the percentage of Gag and CD86 double positive MDDC from 0.003 to 3.3 doses (PFU/DC). (C) Linear regression of percentage of double positive CD86 Gag cells versus viral dose (PFU/MDDC). A representative result of 3 independent experiments is shown. (D) Intracellular Gag expression in MDDC observed by fluorescence microscopy. MDDC were infected with MVA-B and MVA (at 10 PFU/MDDC) and at 16 h post-infection were stained with PE-CD86 (red) and fixed-permeabilized and stained with FITC-anti-HIV-Gag (green) antibody and nucleus was stained with Hoechst (blue). These data are representative of two individual experiments.

After MDDC generation, a flow cytometry analysis showed a purity ≥95% and the phenotype observed was characteristic of immature MDDC: mostly negative (>95%) for CD14, 100% strongly positive for HLA-DR, mostly positive for CD40, CD11c, CD209 (DC-SIGN) and CD86, and negative for CD80 and CD83 (data not shown). These immature MDDC were used for infection with MVA and MVA-B. Using immunofluorescence microscopy with anti-vaccinia virus antiserum (α-WR), we first confirmed that at 16 h post-infection at 10 PFU/MDDC there was expression of vaccinia virus proteins in the cytoplasm of MDDC infected with either MVA-B (Figure 1A) or parental MVA (data not shown).

Since MDDC support infection and expression of MVA genes but do not support production of mature virus [17], [20], [41], a dose-response study was performed to find the optimal infective dose for a maximal intracellular expression of HIV-1 genes and a maximal MDDC viability. To assess MVA-B infection in MDDC, flow cytometry analysis of intracellular Gag protein and cell-surface CD86 as a MDDC marker was performed 16 h post-infection at a dose range of 0.003–3.3 or at a single dose of 10 PFU/MDDC (Figure 1B–D). As a negative control of HIV-1 genes expression, MDDC infected with the parental strain MVA were used. As shown in Figure 1B–C, intracellular Gag expression in MDDC (CD86^+^) showed a linear positive response over a viral dose from 0.003 to 0.33 (slope: 96.823, r^2^ = 0.9996), while at higher viral doses, non-linear increases were found (65.3% and 75.4% at 1 and 3.3 viral dose, respectively).

Using immunofluorescence microscopy, intracellular Gag was observed along the cytoplasm of MDDC infected with MVA-B (at a 10 PFU/MDDC dose), while no Gag was detected in MDDC infected with the parental MVA (Figure 1D). Of note, and in agreement with our previous studies of MDDC infected with MVA [18], we observed an apoptotic nucleus morphology in the infected cells at 16 h post-infection with the highest viral doses (10 PFU/MDDC) (Figure 1A and D).

### Effects of MVA and MVA-B infection on the viability and maturation of MDDC

**Figure 2:**
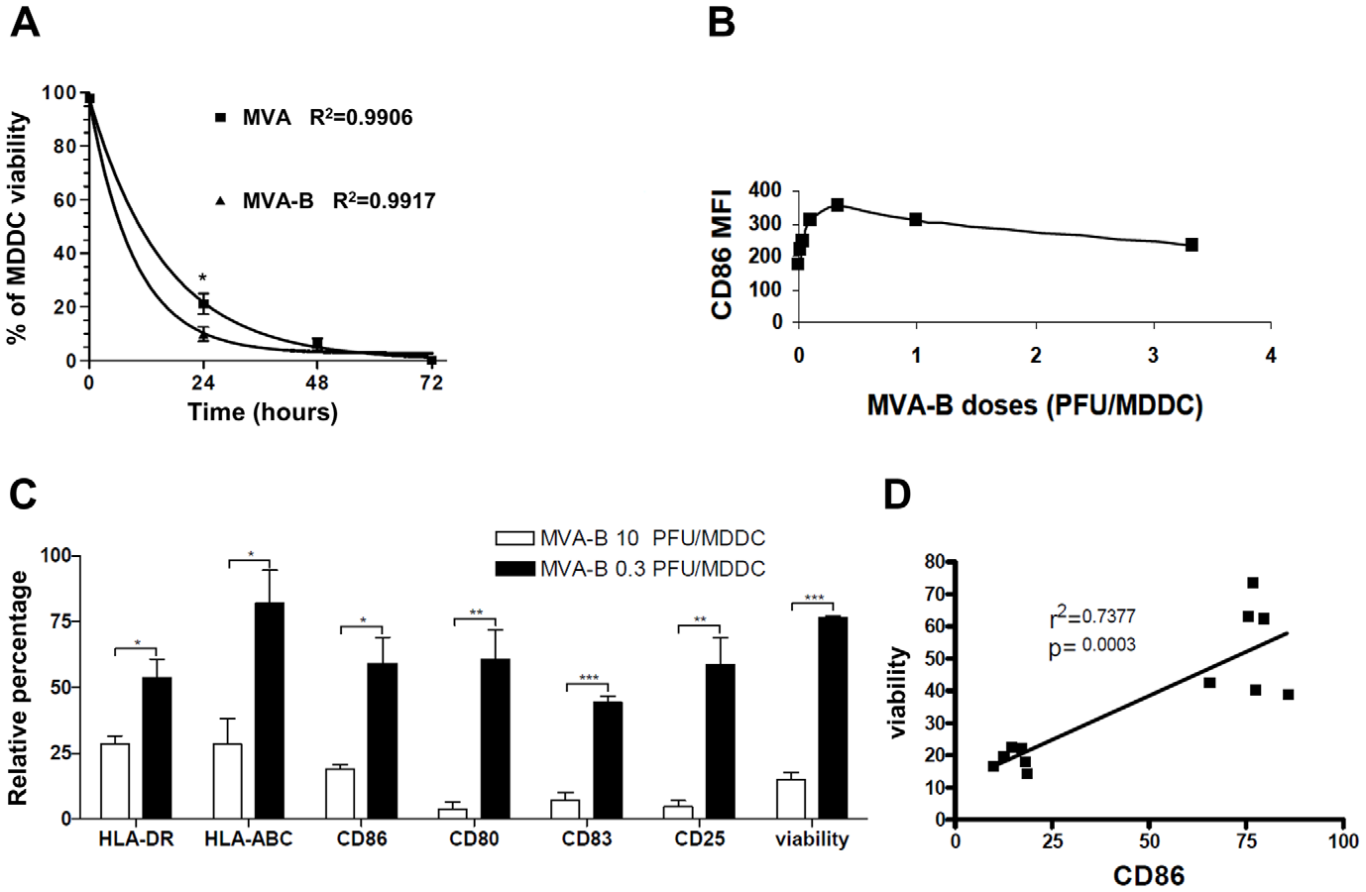
Evaluation of MDDC maturation and viability. (A) Viability assay after MVA and MVA-B infection. Viability was expressed as the percentage of live cells in front of the total basal population. This experiment was performed in triplicates. (*) p<0.05 (B) At 16 h post-infection, in the absence of maturation cocktail, CD86 expression was measured as MDDC maturation surface marker by FACS. CD86 (measured MFI) is represented in a dose-response graphic versus PFU/MDDC at a dose range of 0.003–3.3 PFU/MDDC. This is a representative result of 2 independent experiments. (C) Expression of maturation markers and viability after MVA-B infection at 0.3 and 10 PFU/MDDC. The maturation cocktail was added during 48 h. Maturation markers and viability were measured at 48 h post-infection. HLA-DR, HLA-ABC, CD86, CD80, CD83, CD25 expression was measured as DC maturation surface markers by FACS. Viability was measured by dual Annexin V and 7AAD staining. Live cells were negative for both markers, dead cells were positive for 7AAD and apoptotic cells were positive for Annexin V and negative for 7AAD. Results are represented as the relative percentage calculated with respect to the value obtained with uninfected MDDC: (value of infected MDDC/value of uninfected MDDC)×100. The value was MFI from HLA-DR, HLA-ABC and CD86 or percentage of CD80, CD83, CD25 and viability. (*) p<0.05, (**) p<0.01, (***) p<0.005. Error bars represent the standard error (SEM) from the mean of the replicates (n = 3). (D) Correlation between relative percentage of viable cells and relative percentage of CD86.

In previous studies, we [27] and other researchers [17], [20] had shown that MVA infection of immature human MDDC caused extensive cytopathic effects, particularly at late times post-infection and at high viral doses, that lead to rRNA breakdown and apoptosis [18]. Thus, we next sought to precisely characterize the effects on the MDDC viability following MVA-B infection. First, a time-course measurement of cell viability was performed by flow cytometry defining viable cells as negative for both Annexin V-FITC and 7AAD. As shown in Figure 2A, the viability of MDDC infected with MVA-B at a MOI of 10 PFU/MDDC decreased as a function of time, being virtually null at 72 h post-infection, 5% at 48 h post-infection and 10% at 24 h post-infection. The viability decay analysis with doses of MVA-B of 1 and 3.3 PFU/MDDC gave similar results to those shown in Figure 2A (data not shown). It should be noted that infection of MDDC with MVA resulted in a similar pattern of viability decay, although it was significantly slower than that caused by MVA-B, being the number of viable MVA-infected MDDC 2-fold higher than that of MVA-B-infected MDDC at 24 h post-infection (p<0.05) (Figure 2A). This finding suggests that the inserted HIV-1 genes in the MVA-B vector are somehow involved in causing a higher cytopathic effect compared with the parental MVA.

We next assessed the effect of infection with MVA and MVA-B on the maturation state of MDDC. In a dose-response analysis of CD86 expression and MVA-B dose, at 16 h post-infection, in the absence of maturation cocktail, 0.3 PFU/MDDC was found to be the optimal dose at which CD86 expression assessed as MFI was maximal (Figure 2B). This upregulation of CD86 expression observed at 16 h post-infection at 0.3 PFU/MDDC dose was also coupled with the increase, compared with mock-infected MDDC, of the maturation markers CD80 (from 5.0% to 20.0%), CD83 (from 6.0% to 13.3%), and HLA-DR (from 129.8 MFI to 167.0 MFI) (data not shown). Similar results were obtained for MDDC infected with MVA (data not shown). These data demonstrate that infection of MDDC with both MVA and MVA-B is able to induce by itself a moderate degree of the MDDC maturation, in the absence of any exogenous maturation agents.

Given that, as indicated above, viral doses of MVA-B higher than 1 PFU/MDDC caused a high cell death rate of MDDC at 48 h post-infection, while 0.33 PFU/MDDC at 16 h post-infection appeared as the optimal dose for the upregulation of CD86 and other maturation markers of MDDC, we next compared the highest viral dose, 10 PFU/MDDC, and the optimal dose, 0.33 PFU/MDDC for the effects on maturation and cell viability. These parameters were assessed at 48 h post-infection in the presence of complete maturation cocktail. Although MVA-B infection by itself induces a moderate degree of MDDC maturation, we opted to add the cytokine maturation cocktail after MVA-B infection to obtain the highest degree of maturation, which is a requisite to achieve the highest HIV antigen presentation to T cells [42]. As shown in Figure 2C, on MDDC infected with MVA-B at 0.3 PFU/MDDC dose, the expression of all maturation markers was between 2- and 15-fold higher than MDDC infected with 10 PFU/MDDC dose, with statistically significant differences being observed for all measured markers (CD80, CD83, CD25 and CD86, and HLA-ABC and HLA-DR), expressed as a relative percentage with respect to the maximal value obtained with cocktail-matured uninfected MDDC. MDDC viability, measured by dual staining with Annexin V and 7AAD, was also significantly higher (more than 5-folds) at 0.3 PFU/MDDC than at 10 PFU/MDDC (0.3: 76.57±0.5295, 10: 15.02±2.607, p<0.0001). Moreover, as shown in Figure 2D, a positive linear correlation was observed between MDDC viability and CD86 expression after MVA-B infection and cocktail-induced maturation (r^2^ = 0.7377). The same experiments performed in parallel with the parental MVA showed similar results as those found for MVA-B (data not shown), indicating that the maturation effect at 48 h post-infection is dependent on the MVA vector itself. On the other hand, taken together, the above results suggest that those MDDC that become infected are going to die in a 48 h period, while bystander uninfected and viable cells are those able to undergo the maturation.

### Infection of MDDC with MVA-B induced the production of cytokines, chemokines and IFN-α by MDDC

**Figure 3:**
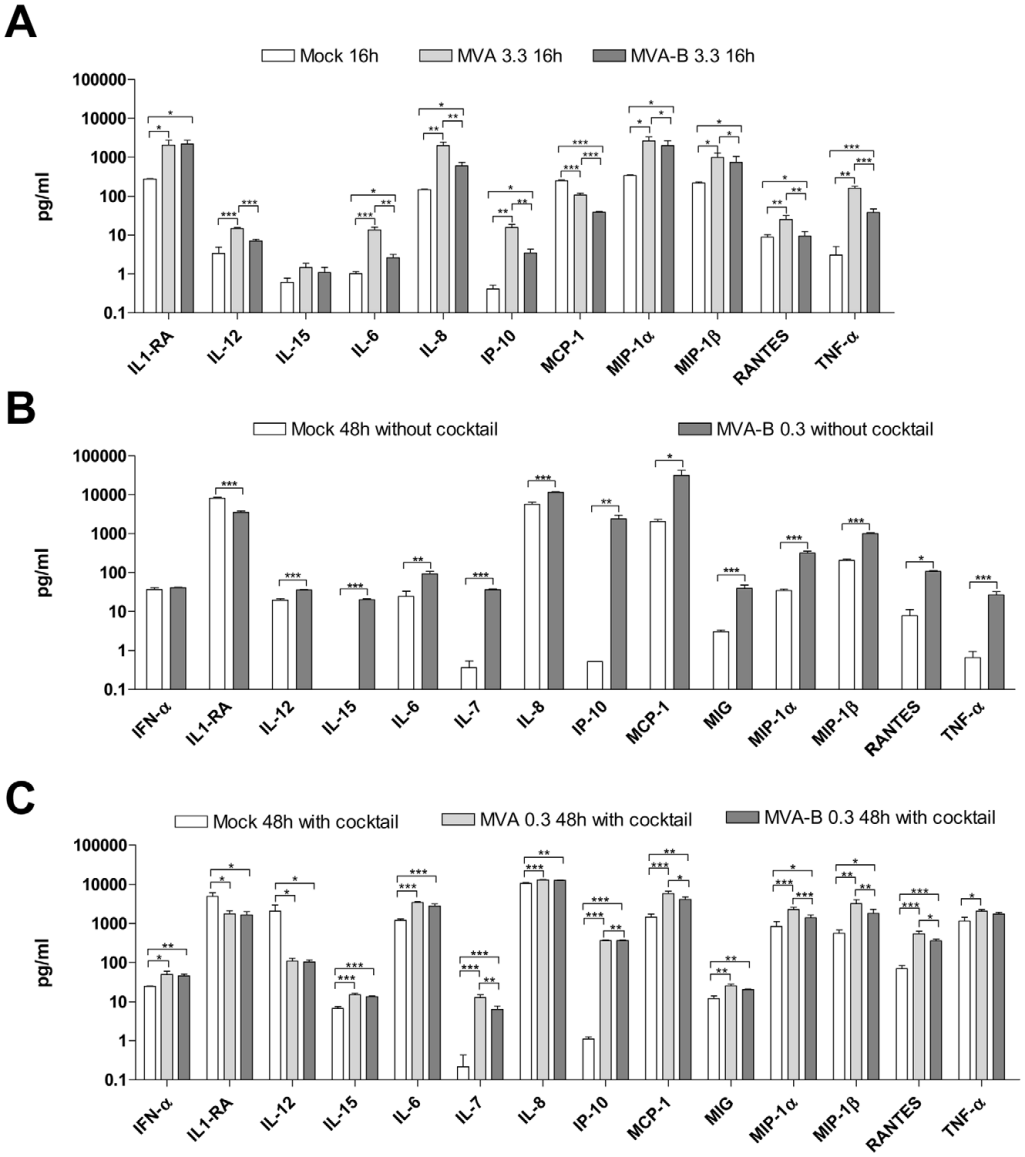
Cytokine, chemokine and IFN-α production by MDDC upon infection with MVA and MVA-B. MDDC were infected with MVA or MVA-B at high doses (3.3 PFU/MDDC) at 16 h post-infection (Figure 3A) and at low doses (0.33 PFU/MDDC) at 48 h post-infection (Figure 3B) and at 48 h post-infection in the presence of maturation cocktail (Figure 3C) and secreted mediators were assessed by Luminex technology. Results are expressed as mean ± SD from 4 independent experiments. Error bars represent the SEM from the mean of the replicates (n = 4). (*) p<0.05, (**) p<0.01, (***) p<0.005.

To further characterize the effects of the infection of MVA and MVA-B in the biology of MDDC, the levels of secreted cytokines and chemokines in the culture supernatants of MDDC following infection with MVA and MVA-B were assessed using the Luminex technology. Cytokine levels secreted by MDDC following infection with MVA and MVA-B were assessed under three conditions: at the high dose of 3.3 PFU/MDDC, after 16 h of infection (Figure 3A), at the low dose of 0.33 PFU/MDDC at 48 h post-infection (Figure 3B) and at 0.33 PFU/MDDC after 48 h of infection but in the presence of maturation cocktail (Figure 3C). MVA and MVA-B induced the secretion of pro-inflammatory cytokines (IL-6 and TNF-α) and chemokines (IL-8/CXCL8, MIP-1α/CCL3, MIP-1β/CCL4, RANTES/CCL5 and IP-10/CXCL10) by the infected MDDC under all the three conditions (Figure 3A, B and C). Interestingly, IL1-ra was also up regulated with MVA-infected MDDC at 16 h post-infection but lower levels were found, compared with mock, at longer periods of time (Figure 3C). MVA and MVA-B also induced secretion of IL-12, but when full maturation was induced, uninfected MDDC also secreted higher amounts of IL-12 (Figure 3A, B and C). After 48 h of infection (Figure 3B and C), MVA-B induced higher production of IL-7, IL-15, MCP-1/CCL2 and MIG/CXCL9 than uninfected MDDC, indicating that some mediators are secreted at later times post-infection and/or at low doses possibly because is necessary some degree of maturation to up regulate such mediators. Similar results were obtained with MVA (Figure 3C, data not shown in Figure 3B). In particular, the levels of IL-7 and IL-15 obtained after MVA and MVA-B infection were approximately 100 fold higher than after mock-infection without maturation cocktail. Statistically significant higher levels for most of the mediators analyzed were observed in MVA versus MVA-B infection (Figure 3A and C). These results suggest that the inserted HIV genes from MVA-B are somehow involved in inducing lower secretion of cytokines comparing with MVA, perhaps as a result of the higher induction of apoptosis of MVA-B (Figure 2A). These results also suggest that a positive correlation between viability and cytokine secretion exists. In Figure 3A, further evidences of this correlation are provided: at 16 h post-infection, differences between MVA and MVA-B are more pronounced (the levels of 9 out of 11 cytokines were significantly higher with MVA-infected MDDC) when the viability differences between MVA and MVA-B were higher (Figure 2A). On the contrary, at 48 h post-infection, when the percentage of viable cells was more similar (Figure 2A) differences between MVA and MVA-B were lower (Figure 3C, the levels of 6 out of 14 were higher with MVA-infected MDDC). Finally, MVA and MVA-B up regulated IFN-α, as shown in Figure 3C.

### MVA-B-infected MDDC induced HIV-1-specific CD8^+^ T cell proliferation and secretion of CD8-polyfunctional-related cytokines in autologous T lymphocytes

**Figure 4:**
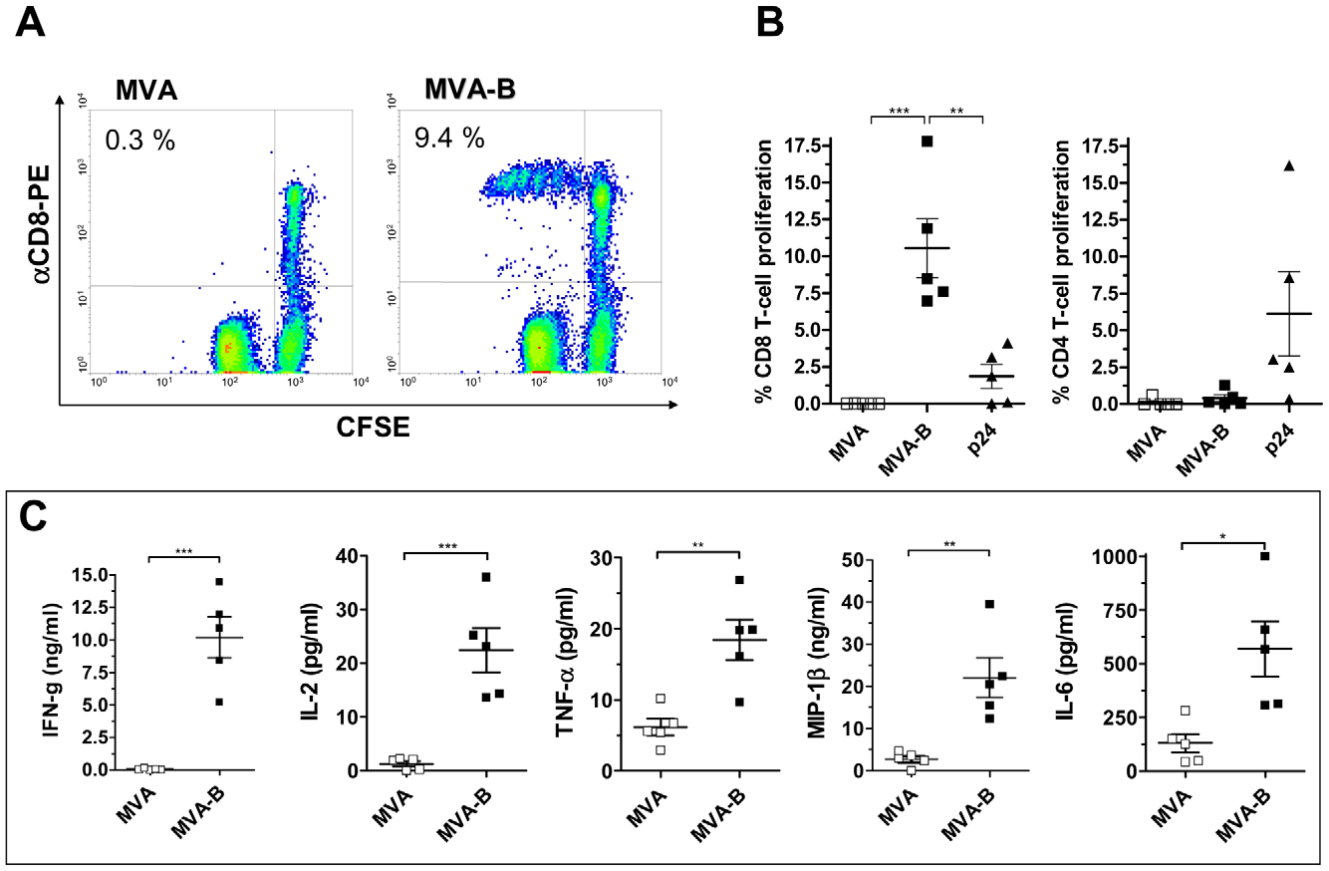
HIV-1-specific T-cell proliferation and cytokine secretion induced by autologous MDDC infected with MVA-B. T cell proliferation in response to autologous MDDC infected with MVA-B at 1 PFU/MDDC in a 6-day co-culture was assessed in triplicates using the CFSE proliferation assay. In (A), a representative (out of 5 independent experiments) flow cytometry plots showing CFSE dilution in gated CD3^+^ CD8^+^ T lymphocytes after *in vitro* stimulation with MVA- and MVA-B-infected MDDC. In (B), the % of CFSE^low^ cells in the CD3^+^ CD8^+^ and CD3^+^ CD8^−^ (CD4^+^) gates responding to MVA-, MVA-B-infected and soluble HIV1p24-pulsed MDDC are shown. Error bars represent the SEM from the mean of 5 patients (n = 5). (***) p = 0.005, (**) p<0.01. (C) Cytokine secretion after co-culture of MVA or MVA-B-infected MDDC and CD8^+^ T lymphocytes. Concentrations of IFN-γ, IL-2, TNF-α, MIP-1β and IL-6 are represented. (***) p<0.005, (**) p<0.01, (*) p<0.05. Error bars represent the SEM from the mean of 5 patients.

We next investigated the capacity of autologous MDDC infected with 1 PFU/MDDC of either MVA-B or MVA to induce HIV-1-specific CD4^+^ and CD8^+^ T cell proliferative responses in fresh autologous lymphocytes of asymptomatic HIV-infected patients (n = 5). As shown in Figures 4A and 4B, MDDC infected with MVA-B induced a significant CD8^+^ T cell proliferative response (with a mean percentage of proliferation of 10.6±2.0%) (Figure 4B). Nearly null proliferation of CD4^+^ T cells occurred in response to MDDC infected with either MVA or MVA-B (Figure 4B). Conversely, CD4^+^ T cell proliferation occurred in response to MDDC pulsed with soluble HIV-1 p24 antigen; while this stimulus induced a low level of CD8^+^ T cell proliferation (Figure 4B).

**Figure 5:**
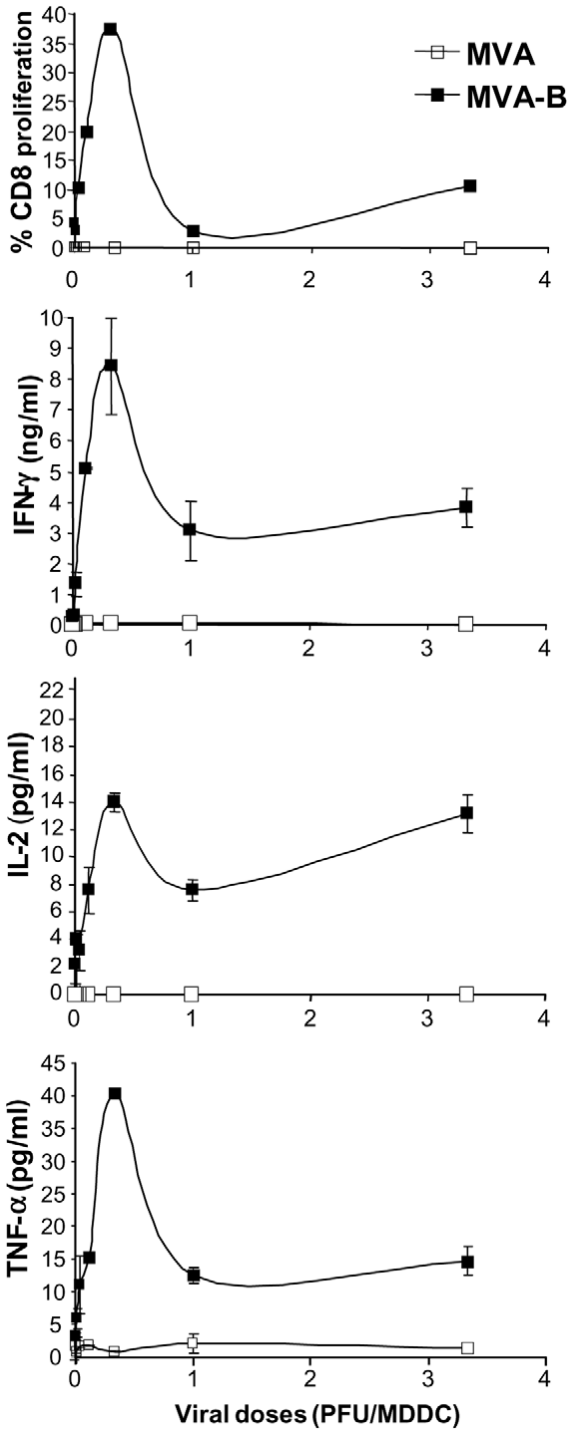
Dose-response analysis of HIV-1-specific T-cell proliferation and cytokine secretion in response to autologous MVA-B-infected MDDC. T cell proliferation and cytokine secretion (IFN-γ, IL-2 and TNF-α secretion) in response to autologous MDDC infected with different doses of MVA and MVA-B (from 0.003 to 3.3 PFU/MDDC) are depicted. A representative result of 2 independent experiments is shown. Error bars represent the SEM from the mean of the replicates (n = 2). Co-culture procedure and methods followed are described in Figure 4.

The proliferative CD8^+^ T cell response to MVA-B-infected MDDC was associated with a high secretion of CD8^+^-polyfunctional-related cytokines as IFN-γ, IL-2, TNF-α, MIP-1β and IL-6 (Figure 4C), and also MIP-1α and RANTES (data not shown). Negligible levels of IFN-γ and IL-2 were secreted in response to MVA-infected MDDC, while MVA-B-infected MDDC induced a high secretion of both cytokines (IFN-γ: 10.19±1.574 ng/ml; IL-2: 22.48±4.10 pg/ml). Secretion of TNF-α, MIP1-β and IL-6 in response to MDDC infected with MVA-B was significantly higher than that found in response to MDDC infected with MVA (MVA: 6.168±1.187 pg/ml, 2.718±0.774 ng/ml and 130.0±43.25 pg/ml, respectively; MVA-B: 18.46±2.804 pg/ml, 22.05±4.704 ng/ml and 569.3±128.0 pg/ml, respectively) (Figure 4C). Similarly, MIP-1α and RANTES levels were significantly higher with MVA-B-infected MDDC (data not shown). In the MDDC alone supernatant only low amounts of TNF-α, IL-6, MIP-1α and MIP-1β were found after MVA and MVA-B infection (data not shown). These values are similar to cytokine levels obtained in the co-cultures after infection with the MVA vector alone (Figure 4C). On the other hand, in the lymphocytes alone supernatant, nearly null levels of cytokines were detected. Moreover, due to the fact that proliferating CD8^+^ T cells secrete such cytokines and, as expected, we have found that CD8^+^ T cells proliferation in the co-culture of MVA-B-infected MDDC positively correlated with these cytokine secretion (Figure 5) and, since CD8^+^ T cells are the main proliferating population (95.65% of total proliferation, Figure 4B), we can assume that the measured cytokines produced in the supernatants of MVA-B infected MDDC co-cultured with lymphocytes mainly correspond to the proliferating CD8^+^ T cells. Only a residual amount would correspond to CD4^+^ T cells (4.35% of the total proliferation, Figure 4B) and only a low secretion of TNF-α, IL-6, MIP-1α, MIP1-β is induced by the infected MDDC alone.

The HIV-1-specific T cell proliferative response and cytokine secretion (IFN-γ, IL-2 and TNF-α) were further assessed in a dose-response study at a MOI range from 0.003 to 3.3 PFU/MDDC. As shown in Figure 5, the CD8^+^ T cell proliferative response showed a MOI-dependent increase to reach a maximum at a concentration of 0.3 PFU/MDDC, from which the response decreased up to a plateau starting at around 1 PFU/MDDC. Thus, 0.3 PFU/MDDC was again found to be the optimal dose for CD8^+^ T lymphocyte proliferation. It should be noted that HIV-1-specific proliferative response of CD4^+^ T cells in these dose-response experiments was also nearly null as it occurred above with the 1 PFU/MDDC dose (data not shown). The dose-response curve in terms of secretion of IFN-γ, IL-2 and TNF-α was similar to that of CD8^+^ T cell proliferation, indicating that a positive correlation exists between CD8^+^ T cell proliferation and secreted cytokines. Indeed, the levels of secreted IFN-γ, IL-2 and TNF-α increased with the dose to reach a peak at around 0.3 PFU/MDDC, from which the response decreased to a constant level when MOI was ≥1 PFU/MDDC. Therefore, as observed for MDDC maturation and CD8^+^ T cell proliferation, 0.3 PFU/MDDC was found to be the optimal dose for cytokine secretion (Figure 5).

**Figure 6:**
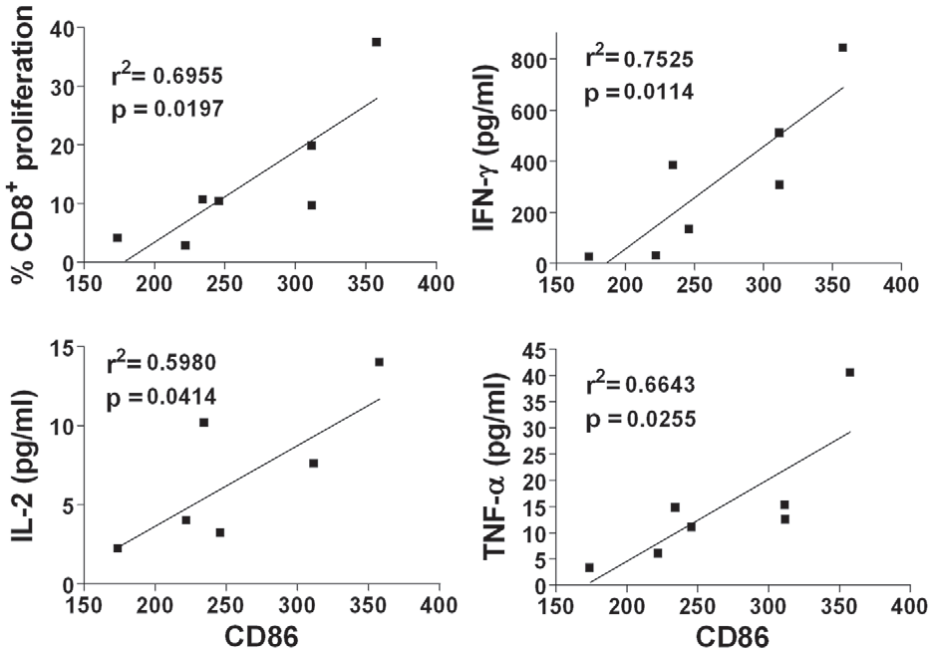
Correlation between CD86 expression on MVA-B-infected MDDC and HIV-1-specific CD8^**+**^ T-cell proliferation and cytokine secretion. MDDC were infected with MVA-B at serial three-fold dilutions (from 0.003 to 3.3 PFU/MDDC) and expression of the maturation surface marker of MDDC, CD86, measured by flow cytometry at 16 h post-infection in the absence of maturation cocktail was assessed. Subsequently, MVA-B-infected MDDC matured with cytokine cocktail were co-cultured for 6 days with autologous lymphocytes and HIV-1-specific CD8^+^ T proliferation and cytokine secretion were measured. A representative result of 2 independent experiments is shown.

The correlation between the maturation degree of MVA-B-infected MDDC and HIV- specific T cell responses was analyzed. With this purpose, MDDC were infected at a dose range of 0.003–3.3 PFU/MDDC and expression of the maturation surface marker of MDDC, CD86, were measured by FACS. Subsequently, infected MDDC were co-cultured with autologous T lymphocytes for 6 days, and the CD8^+^ T lymphocyte proliferation and secretion of IFN-γ, IL-2, and TNF-α was measured as described above. In all cases, a direct and significant correlation was observed between CD86 expression (MFI) and the percentage of CD8^+^ T lymphocyte proliferation and levels of secreted IFN-γ, IL-2 and TNF-α secretion, with correlation coefficients (r^2^) of 0.6955, 0.7525, 0.5980 and 0.6643, respectively (Figure 6).

### CD8^+^ T cells activated by MVA-B-infected MDDC showed cytotoxic activity against autologous HIV-infected CD4^+^ T cells

**Figure 7:**
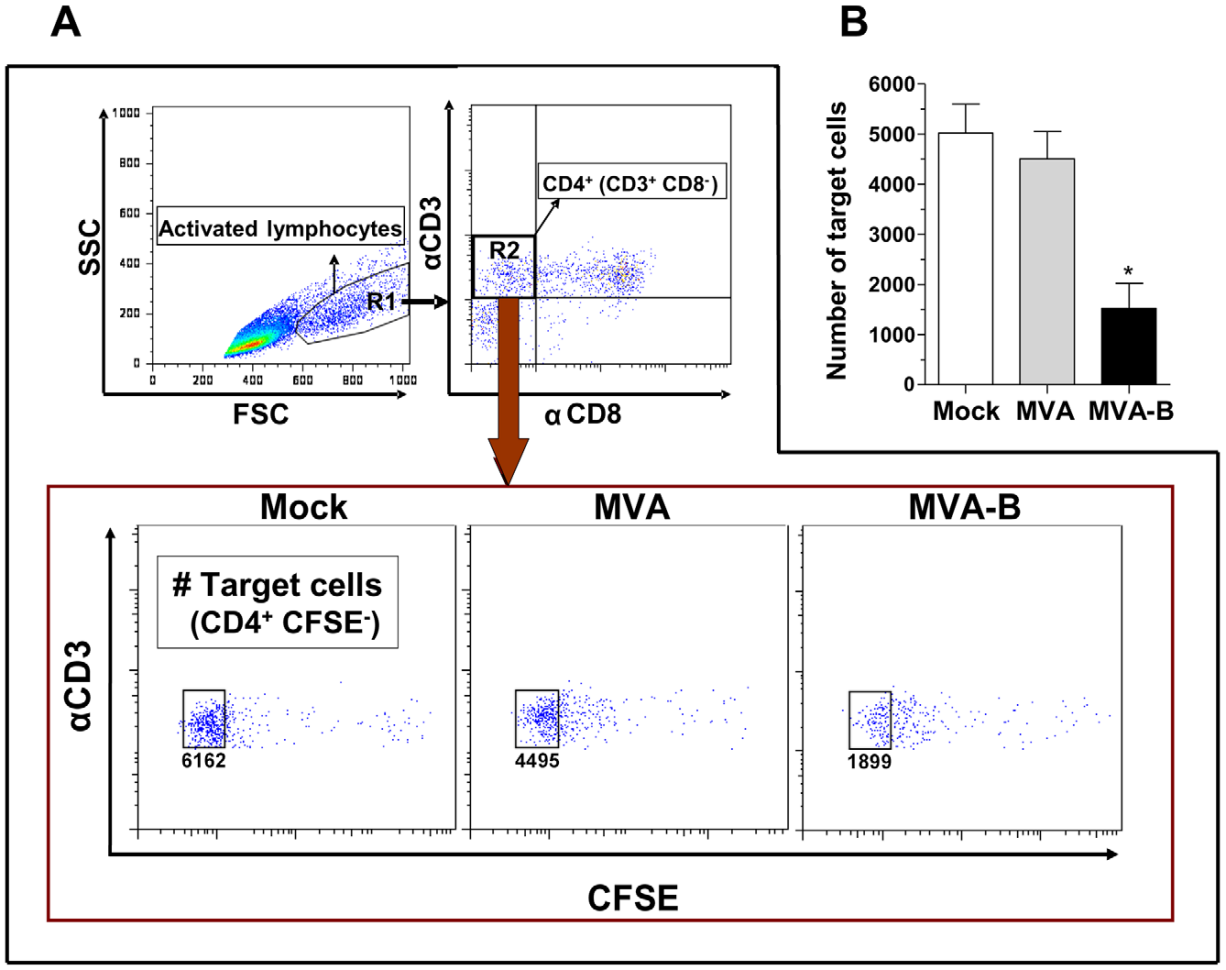
Cytotoxic activity of CD8^+^ T lymphocytes activated by MVA-B-infected MDDC. Effector cells were autologous monocyte-depleted PBMC co-cultured for 6 days with MDDC mock-treated or infected with MVA or MVA-B. After this co-culture period, CFSE staining of effector cells was performed and then were cultured for 4 h with target cells at a 10∶1 ratio. Target cells were autologous CD8-depleted T cells infected with a primary HIV-1 isolate and their corresponded to the phenotype CFSE-negative, CD3-positive CD8-negative. In (A) the flow cytometry analysis is shown. In (B), the mean (±SEM) count of target CD4^+^ T cells is a representative result of three independent experiments (performed in triplicates) is depicted. (*) p<0.05.

After co-culture for 6 days with MVA-B-infected MDDC (at a ratio of 0.3 PFU/MDDC) CD8^+^ T lymphocytes showed a strong cytotoxic activity against HIV-infected autologous CD4^+^ T lymphocytes. After a co-culture of 4 hours between stimulated CD8^+^ T cells and HIV-infected CD4^+^ T cells, the CD4 count showed a 69.8% of reduction, from a mean of 5.02×10^3^ cells to 1.52×10^3^ cells when MVA-B was used. By contrast, CD8^+^ T cells stimulated with MDDC infected with the parental strain (MVA) showed a slight cytotoxic activity with a mean of 10.4% of reduction (from 5.02×10^3^ to 4.5×10^3^), with statistically significant differences being obtained between MVA and MVA-B (p = 0.0160) (Figure 7B).

### MVA-B-exposed MDDC after induction of fully maturation were able to migrate toward a chemokine gradient

**Figure 8:**
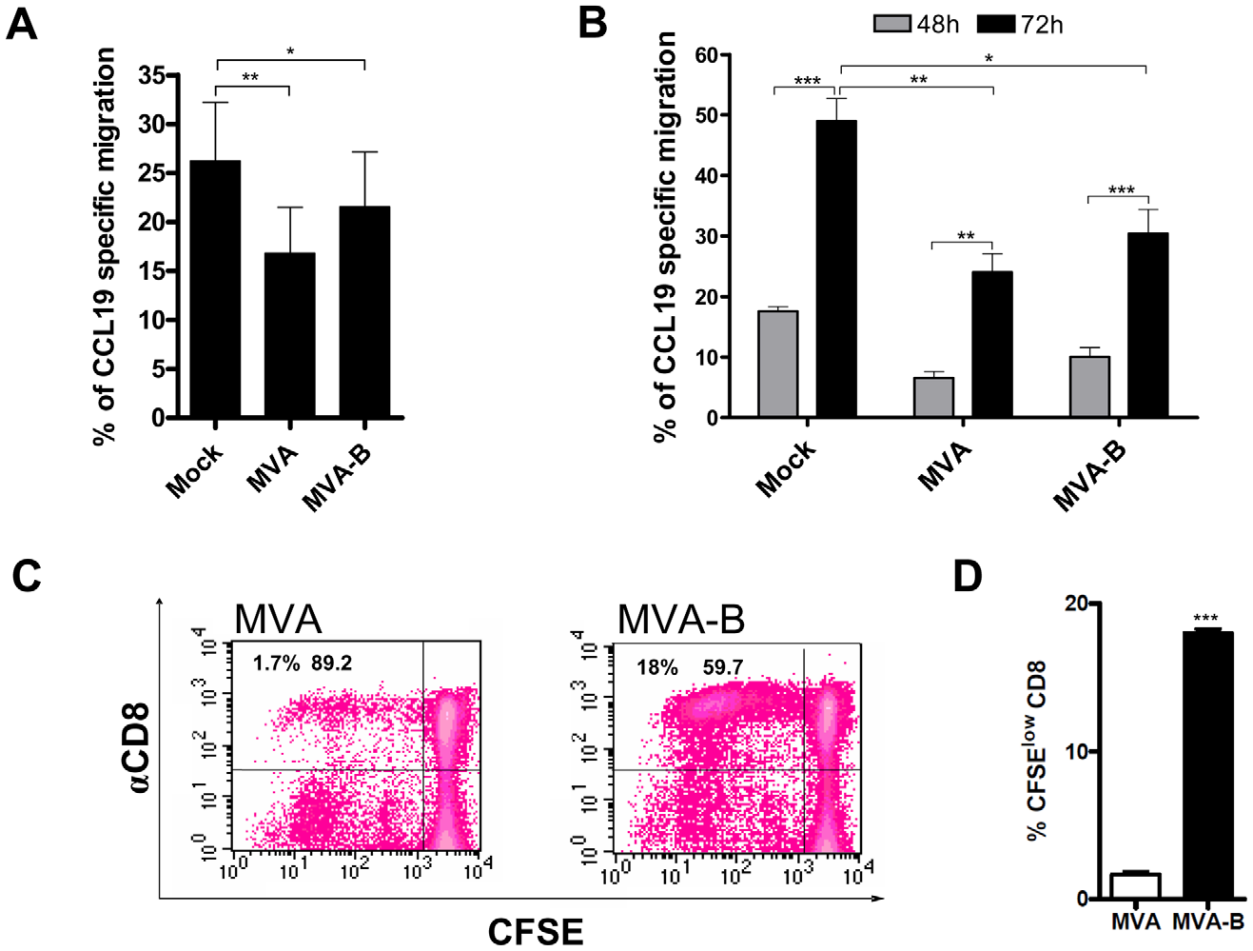
Chemotaxis assay. Chemokine-induced migration of infected mature MDDC was tested 48 h and 72 h after infection in a chemotaxis assay (n = 6). Entire populations that migrated toward CCL9 and CCL21 were collected after incubation and counted during one minute by flow cytometry. The number of migrated DC is depicted in percent of the initial input. Subsequently, infected (MVA and MVA-B) and uninfected MDDC that migrated in the chemotaxis assay were collected and co-cultured with CFSE labeled autologous lymphocytes at a 1∶40 ratio (5×10^3^ MDDC: 2×10^5^ lymphocytes/well). After a period of 6 days, proliferation was tested by flow cytometry. (A) Percentage of CCL19 specific migration of MVA-B-infected MDDC 48 hours after infection. Error bars represent the mean (±SEM) from 6 independent experiments with 6 different HIV-1 patients (n = 6) performed in triplicates. (B) Percentage of CCL19 specific migration of MVA and MVA-B-infected and uninfected MDDC (Mock) 48 and 72 h post-infection and maturation. Error bars represent the mean (±SEM) from 3 independent experiments with 3 different HIV-1 patients (n = 3) performed in triplicates. (C) Representative flow cytometric plots showing CFSE dilution in gated CD3^+^ CD8^+^ lymphocytes after co-culture with MVA and MVA-B infected MDDC that were able to migrate in the chemotaxis assay. One representative result of 2 independent experiments is shown. Error bars represent the mean (±SEM) (n = 3). (D) Percentage of CFSE^low^ cells in the CD3^+^ CD8^+^ gate, observed in one sample. Error bars represent the SEM from the mean triplicates (***) p = 0.005.

It is known that MVA inhibits directional migration of mature DC toward the lymphoid chemokines CCL19 and CCL21 without affecting surface expression of the CCR7 chemokine receptor or impairing undirected cellular locomotion [43]. For this reason, we assessed the influence of MVA and MVA-B infection (at 0.3 PFU/MDDC) on the capacity of infected mature MDDC to migrate toward a gradient of chemokines CCL19 and CCL21. Both uninfected (mock) and MVA- and MVA-B-infected MDDC were matured for 48 h with the maturation cytokine cocktail. As shown in Figure 8A, where the results of six experiments with cells from different patients are depicted, compared with mock-infected MDDC (25.21%), both MVA and MVA-B infections reduced the migration of MDDC at 48 h post-infection (16.82% for MVA and 21.53% for MVA-B). However, MVA-B infection inhibited MDDC migration to a lesser extent than MVA did, although this difference did not reach statistical significance (21.53%, p = 0.029 vs negative control and p = 0.261 vs MVA). Similar results were also obtained in the same migration assay using chemokine CCL21 (data not shown). On the other hand, migration evaluation performed at 48 h and 72 h post-infection and maturation with uninfected (Mock) and MVA- and MVA-B-infected MDDC showed a clear increase, higher than 2-folds, in the number of migrated cells at 72 h compared with 48 h (in MVA-B-exposed MDDC migration increased from 10.05% at 48 h to 30.38% at 72 h, p = 0.0005) (Figure 8B). These results indicated that longer periods of maturation would induce a higher degree of migration.

We also analyzed whether MDDC that had been exposed to both MVA and MVA-B and had migrated to CCL19 were capable of inducing lymphoproliferative responses. For this purpose, migrated cells were collected and immediately co-cultured for 6–7 days with fresh autologous T lymphocytes. As shown in Figure 8C, migrated MDDC infected with MVA-B induced specific CD8^+^ T cell proliferation, with a proliferation rate 10-fold higher than that obtained with MDDC pulsed with MVA (18.0% vs 1.7%, p<0.0001, Student's t test) (Figures 8C and 8D).

### Upon MVA-B infection, MDDC underwent apoptosis and were phagocytosed by uninfected bystander MDDC that cross-presented HIV-1 antigens to CD8^+^ T lymphocytes

**Figure 9:**
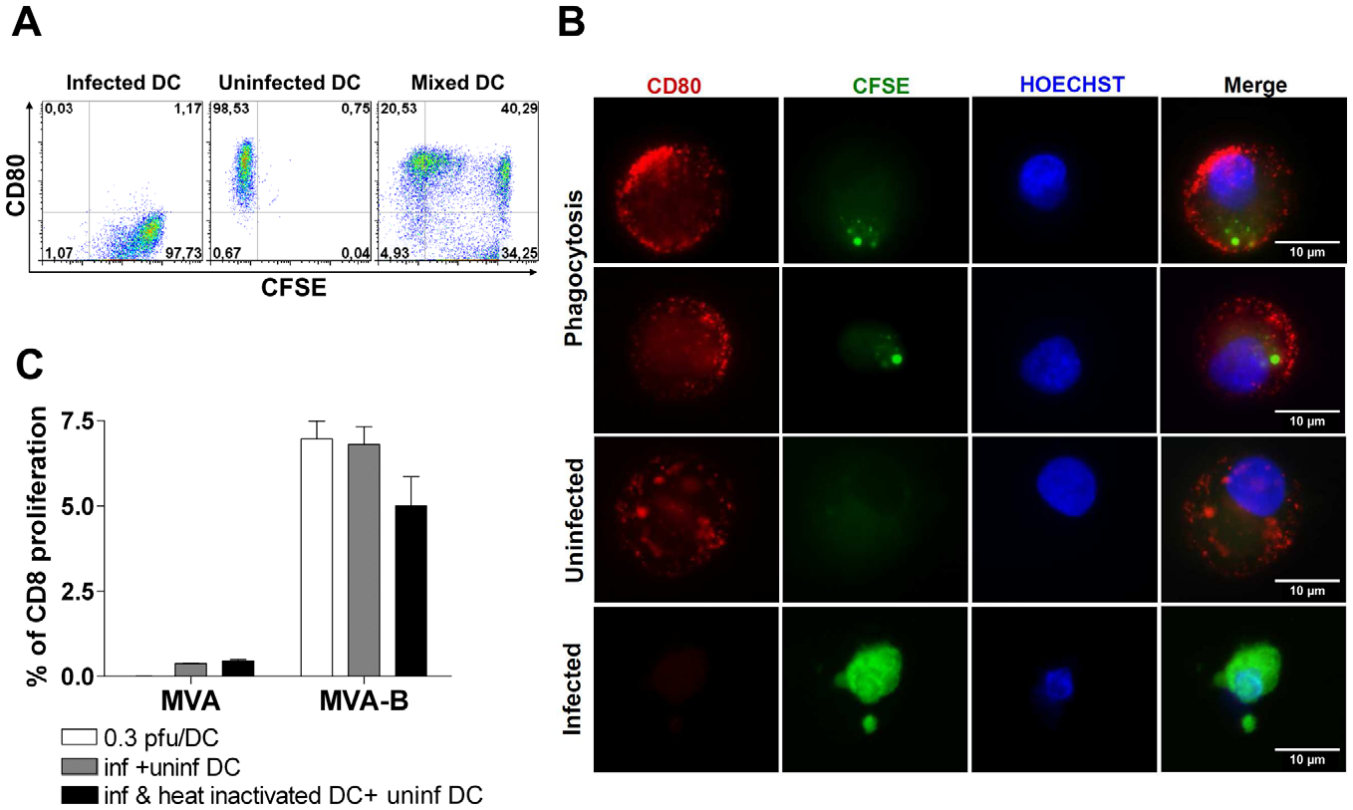
Antigen presentation study. (A) Phagocytosis assay. Immature MDDC were labeled with CFSE and infected with MVA at a MOI of 10 PFU/MDDC for 1 hour. They were then extensively washed and mixed with uninfected immature MDDC (ratio 1∶2) and maturation was induced for 48 h. Phagocytosis of apoptotic bodies resulting from MVA-infected MDDC by uninfected MDDC were detected by flow cytometry. Density plots showing infected MDDC, detected as CFSE^+^ CD80^−^ (*left box*), uninfected MDDC, detected as CFSE^−^ CD80^+^ (*middle box*) and CD80^+^ MDDC that ingested apoptotic CFSE-labeled MDDC during the incubation period, detected as CFSE^+^ CD80^+^ (*right box*). These data are representative of two individual experiments. (B) Phagocytosis assessed by fluorescence microscopy. Phagocytosis of apoptotic bodies resulting from MVA-infected MDDC by uninfected MDDC were visualized by fluorescence microscopy. MVA-B infected MDDC were CFSE^+^ CD80^−^ (green colour), uninfected matured MDDC were stained CFSE^−^ CD80^+^ antibody (red colour), nucleus was visualized with Hoechst staining (blue colour) and phagocytosis was observed as CFSE stained vacuoles (green) inside the MDDC CD80 positive cells (red). As negative controls of phagocytosis, uninfected MDDC and infected MDDC cultured separately were used. These data are representative of 2 independent experiments. (C) Cross-presentation induced proliferation of CD8^+^ T cells. Representative dot plots showing the proliferation rate of CD8^+^ T cells after co-culture between infected MDDC with autologous lymphocytes. The white plots correspond to a MVA-B infection model at 0.3 PFU/MDDC. The black plots correspond to infection of immature MDDC (two thirds of the initial culture; cultured during 72 hours) with apoptotic bodies (obtained after infection of MDDC –one third of the initial culture- with MVA-B at a dose of 10 PFU/MDDC and during a period of 72 hours). The grey plots correspond to infection between immature MDDC and apoptotic bodies obtained as described above and subsequently heat-inactivated at 55°C for one hour. Subsequently, infected MDDC were collected and co-cultured with CFSE labeled autologous lymphocytes in triplicates. Six days after, proliferation was tested by flow cytometry. These data are representative of 3 individual experiments.

The cytopathic effects of MVA-B infection (see Figure 2A and 2C) and the capacity of viable, mature and migrated MDDC to induce the proliferation of HIV-specific CD8^+^ T lymphocytes documented above suggest that these cells correspond to those MDDC that captured apoptotic MVA-infected MDDC and then cross-presented HIV antigens to HIV-specific CD8^+^ T cells. To test this hypothesis we first evaluated the capacity of uninfected MDDC to engulf the apoptotic bodies resulting from MVA-B infection on MDDC. To this end, CFSE-labeled immature MDDC were infected with MVA and MVA-B at a MOI of 10 PFU/MDDC for one hour and then mixed with uninfected immature MDDC at a ratio of 1∶2 (infected∶uninfected) and incubated with the maturation cocktail for 48 h. Afterward, staining of cell-surface CD80 was performed and then phagocytosis of MVA-B-infected MDDC by uninfected MDDC was assessed by flow cytometry considering the phenotypes CFSE^+^ CD80^−^ (dead MVA-B-infected MDDC), CFSE^−^ CD80^+^ (uninfected matured MDDC), CFSE^+^ CD80^+^ (CD80^+^ uninfected matured MDDC that ingested apoptotic CFSE^+^ MDDC during the incubation period). As depicted in Figure 9A, the percentage of MDDC that ingested apoptotic bodies was 40.3%. These results were also confirmed by fluorescence microscopy, where phagocytosis was indicated by the localization of apoptotic cells or apoptotic bodies (CFSE, green colour) within CD80-positive MDDC (PE, red colour) (Figure 9B).

As indicated above, at a concentration of 0.3 PFU/MDDC, a maximum response was obtained not only in terms of MDDC maturation and viability, but also in terms of CD8^+^ T cell lymphoproliferative and cytokine secretion responses (Figure 2A, Figures 5A and 5B). Based on all these results, we hypothesized that after MVA-B infection at an optimal dose of 0.3 PFU/MDDC, approximately one third of the MDDC culture is infected, resulting in their apoptosis and converted into apoptotic bodies, while approximately the other two thirds of the initial mature MDDC would remain uninfected, being able to engulf the cell debris containing MVA-B-mediated HIV-1 expressed antigens and to maturate properly and, thus, cross-present the HIV antigens to CD8^+^ T lymphocytes. Results obtained clearly support this hypothesis. Indeed, as can be seen in Figure 9C, similar HIV-1-specific CD8^+^ T cell lymphoproliferative responses were obtained in the model of optimal infection (0.3 PFU/MDDC) and in a model where a ratio of 1∶2 (apoptotic bodies: uninfected MDDC) was used, with no significant differences between both experiments (optimal dose: 6.97% of CD8^+^ proliferation; apoptotic bodies: 6.81% of CD8^+^ proliferation, p = 0.8383). Of note, additional experiments in which the apoptotic bodies were heat-inactivated at 55°C for one hour resulted in a similar proportion (5.00%) of HIV-1-specific CD8^+^ T cell proliferation. These results indicate that the HIV-1-specific CD8^+^ T cell proliferation induced by MVA-B-infected MDDC is mainly due to cross-presentation of HIV-1 antigens.

## Discussion

After the failure of the adenovirus-based anti-HIV vaccines at phase III clinical studies 2008 [10]–[12] and with the publication in 2009 of the first promising results of a prophylactic anti-HIV vaccine based on poxviruses vectors [13], basic and clinical research with poxviruses such as MVA has notably increased. Since MVA-B is currently being evaluated in phase I clinical trials and due to the essential role of MDDC in the development of an efficient immune response, the present study investigated, in an *ex vivo* model, the impact of MVA-B infection on the biology of MDDC from asymptomatic HIV-1-infected patients, and the capacity of MVA-B-infected MDDC of these patients to induce HIV-1-specific T cell responses in autologous T lymphocytes. In that setting, another more specific goal was to assess whether MVA-B could be used as an immunogen in a DC-based effective therapeutic HIV-1 vaccine. The *in vitro* model using autologous fresh MDDC from asymptomatic HIV-infected patients is considered a more realistic scenario to support the efficacy of these type of therapeutic vaccines (where HIV could have influenced on MDDC biology), in comparison with other models using MDDC from healthy volunteers [20].

Data obtained in the present study demonstrate that MVA-B-infected MDDC from HIV patients express Gag intracellular protein, consistently with our previous works with MDDC from healthy subjects [44], and with other recent studies using other MVA vectors carrying HIV-1 genes [20]. Our data revealed that the expression of intracellular Gag was dose-dependent and we also found that MVA and MVA-B infection produced a rapid cytopathic effect on the infected cells, being this phenomenon associated with direct infection of the cells. These results are consistent with previous reports using MDDC from healthy individuals infected with MVA vectors expressing HIV antigens [17], [18], [20]. In our study, we extended infection up to 72 hours, when we observed a nearly null percentage of viable cells infected with both MVA and MVA-B. Of note, MVA-B induced a more rapid apoptosis than MVA, in agreement with other reports using another HIV-1-MVA construct [17]. In this context, HIV proteins expressed by MVA-B could be involved in this quicker induction of apoptosis. We also found that MVA and MVA-B infection induces MDDC maturation in a dose-dependent manner, being maximal at 0.3 PFU/MDDC, may be due to the fact that infected MDDC secreted TNF-α, IL-6 and type I IFN, which are known to induce DC maturation [42], [45]–[47]. Interestingly, at this dose, uninfected bystander DC in contact with MVA or MVA-B-infected cells underwent phenotypic maturation without apoptosis and without any maturation factor added, in accordance with studies in MDDC from healthy volunteers and in murine DC [20], [48]. In agreement with these previous works, at higher MOIs, viability was drastically reduced and we found that it was coupled with a marked decrease of maturation markers.

To advance in the knowledge of cytokine secretion from previous studies [20], where production of IFN-α was assessed, we have analyzed the production of an array of 25 cytokines by MDDC of asymptomatic HIV-1-infected patients after infection with MVA and MVA-B, and we found that the secretion of 13 of such mediators (IFN-α, IL1-ra, IL-12p40/p70, IL-15, IL-6, IL-7, IL-8, IP-10/CXCL10, MCP-1/CCL2, MIG/CXCL9, MIP-1α/CCL3, MIP-1β/CCL4, RANTES/CCL5 and TNF-α) was upregulated. These results are in accordance with our previous studies demonstrating that infection with MVA or MVA-B of immature MDDC from healthy individuals upregulated the expression of most genes encoding the above mentioned immunomodulators (*IFNα, IFNβ, TNFα, IL-6, IL12β, CCL3, CCL4, CCL5 and CXCL10*) [18], [44]. Production of most of these cytokines was recently also found in human macrophages infected with MVA [49]. In agreement with our results, recent studies described secretion of IFN-α [20], [43] and we have also previously shown secretion of IFN-β [44] in human MVA-infected MDDC. IFN-α is involved of upregulation of inflammatory chemokine receptors such as CCR1, and CXCR1 by uninfected bystander DC [43]. On the other hand, we also showed that MVA or MVA-B-infected cells secrete high amounts of MCP-1, CCR5 and CCR1 ligands such as MIP-1α, MIP-1β and RANTES that would promote the attraction of uninfected MDDC to the inflammation site [43], [50], where they can engulf the apoptotic infected MDDC. Interestingly, these CCR5-chemokines inhibit HIV-1 infection of CCR5-tropic (R5) HIV-1 isolates [51], suggesting that MVA-infected DC would inhibit HIV infection. In this context, IFN-α is also known to exert important antiviral effects and exogenously added IFN-α has been shown to inhibit HIV replication [20], [52] and to limit HIV cell-to-cell spread [20], [53]. Consistent with the IFN-α secretion observed after MVA and MVA-B infection of MDDC, the present study also described secretion of MIG/CXCL9, which, together with IP-10/CXCL10, is known to be markedly upregulated in the presence of IFN-α [54]. MIG and IP-10 stimulate the directional migration of activated T cells, thus contributing to induce HIV-specific CTL responses [55]. Importantly, we demonstrate for the first time that MDDC infected with MVA and MVA-B, secrete IL-7 and IL-15 at levels more than 30-fold higher than the negative control after 48 h post-infection without maturation cocktail. IL-7 and IL-15 were detected previously after MVA infection of HeLa cells [27]. IL-7 is a T cell homeostatic cytokine [56] that regulates the ability of T cells to interact with DC, thereby influencing both T cell priming and homeostasis [57]. In a recent study, IL-7 secretion has been upregulated in CD40L-mature MDDC, having a relevant role as a “costimulator” cytokine during DC/antigen activation leading to an increased expansion of antigen-specific CD8^+^ T cells [58]. Of note, our work has reported IL-7 levels about 10-fold higher than those obtained in this previous study [58], which is consistent with the high HIV-specific CD8^+^ T cell proliferation observed, which would be enriched in long memory CD8^+^ T cells due to the fact that IL-7 and IL-15 are also upregulated [59], [60]. The present study also showed secretion of IL-8 chemokine. Interestingly, in a recent study of SHIV infection, higher plasma levels of IL-8 were associated with lower susceptibility to infection [61], presumably due to the neutrophil attractant activity of IL-8 [61]. We speculate that this activity would stimulate defensin-1 and defensin-2 release [62], which in immature MDDC have been associated with slower disease progression in HIV patients as we have recently demonstrated [63]. Regarding IL-12, secreted levels were higher upon MVA and MVA-B infection than in uninfected MDDC. Previous results suggest that MVA specially induce IL-12p40 (*IL12β* gene) [18], [49], thus allowing MDDC to maturate and subsequently to develop an enhanced CD8^+^ T cell response [64], [65], which is recommended for a vaccine against HIV-1 infection [66], [67].

Since MDDC pulsed with MVA-B is currently being developed to be tested in clinical trials, one of the main aims of this study was also to find the optimal MOI at which MVA-B-infected MDDC activate strong HIV-specific immune responses. The dose of 0.3 PFU/MDDC was found to be the optimal one for MDDC viability, maturation and high levels of Gag-antigen expression, and, accordingly, it was also found to be the optimal dose for HIV-1-specific CD8^+^ T cell responses. In this context, we have shown that MVA-B infection of MDDC stimulates a strong HIV-immune response, mainly induced by CD8^+^ T cell proliferation and coupled with a high secretion of CD8^+^-polyfunctional-related cytokines. Such CD8-immunoresponses are considered as an important immune correlate of protection against HIV-1 [68]–[73], more complete than the assessment of IFN-γ by ELISPOT performed in previous studies [20]. In addition, MIP-1α, MIP-1β and RANTES secreted by CD8^+^ T cells would inhibit HIV-1 infection [74], [75]. What is more, for the first time, the present study has evaluated cytotoxic responses induced by MVA-B using HIV-infected CD4^+^ lymphocytes as target cells. Results obtained have clearly shown that MVA-B infection induces a strong cytotoxic activity by stimulated CD8^+^ T lymphocytes (69.8% reduction of target cells), thus demonstrating that the combination of MDDC and MVA-B is able to destroy the main target cells where HIV replicates, which could be used as a direct marker of the efficacy of vaccine candidates [76].

Another important advance of this study in comparison with previous works [20] is the demonstration that after MVA and MVA-B exposure, MDDC are able to migrate toward a gradient to chemokines secreted by lymph nodes. It had been postulated that different strains of vaccinia virus could interfere in DC functions, in particular, inhibiting migration to secondary lymphoid organs [43], [77]. MVA inhibited directional migration of infected and bystander immature DC subsequently matured with LPS for 24 h toward a gradient of CCL19 chemokine [43]. Based on these observations, we also assessed the migration capacity of MDDC infected with MVA and MVA-B and subsequently fully maturated to migrate toward a gradient of chemokines CCL19 and CCL21 (found in lymph nodes and whose receptor -CCR7- is expressed in mature DC) 48 h and 72 h after infection. We observed that migration of MDDC infected with MVA was significantly reduced in comparison with uninfected cells but, in contrast, MVA-B impaired MDDC migration to a lesser degree. Of note, we demonstrated that the fraction of migrated cells maintained their capacity to stimulate HIV-specific-CD8^+^ T lymphoproliferative responses and that the migration capacity after MVA-B infection increased with time, being maximal at 72 h post-infection, when MDDC had fully maturated and, consequently, had increased their capacity to migrate. Previous studies reported a higher impairment of migration not only in MVA-infected MDDC but also in bystander MDDC, maybe due to the lower times of maturation period (migration was evaluated 24 h post-infection) [43], thus probably not giving enough time to MDDC to fully maturate and to undergo upregulation of CCR7. In the observed higher migration capacity of MVA-B-infected MDDC, we think that the expression of HIV proteins could have a role in accelerating apoptosis and inducing a lower proinflammatory chemokine expression (as demonstrated in this study), which would allow a slightly higher degree of maturation by the added maturation cocktail. In any case, results of this study have clearly demonstrated that upon MVA-B infection, bystander and viable MDDC are able to capture HIV antigens and, after full maturation, are able to migrate toward a gradient of CCL19 and CCL21, thus they would be competent to reach secondary lymphoid organs. What is more, migrated MDDC mainly interact with CD8^+^ T lymphocytes and stimulate HIV-specific immune responses. Since the migration capacity toward lymph nodes is an essential function of antigen presenting-cells such as MDDC, we think these results are a surrogate marker of the efficacy of these MVA-B based vaccines that are being tested in clinical trials.

Our results also demonstrated that the induction of CD8^+^ T cell mediated immunity after interaction with MVA-B-infected MDDC mainly depended on cross-presentation, being these results consistent with previous observations [20], [48], [78], [79]. In comparison with previous works in which cross-presentation was reported after contact of MVA-HIV-infected epithelial cells or myotubes with DC [20], the novelty of our study is that we described for the fist time the cross-presentation phenomenon after interaction between two types of MDDC: the apoptotic MVA and MVA-B infected MDDC and the bystander MMDC (of particular relevance for a MDDC based vaccine). The apoptosis observed after MVA and MVA-B infection has been proposed as a reason for its greater immunogenicity, presumably through an enhanced release of nucleotides, HIV-antigen degradation and enhanced cross-presentation to CD8^+^ T cells [80]–[82]. Another explanation for the strong induction of CD8^+^ T cell responses with very low activation of CD4^+^ T cells would be the induced secretion of cytokines by MVA-infected MDDC as Type I IFN [83], IL-7 [58] and IL-12 [64], [65]. IFN-α-stimulated DC can induce cross-presentation to CD8^+^ T cells without the help of CD4^+^ T cells in murine models [83] and immunized hu-PBL-SCID mice with inactivated HIV-1-pulsed IFN-α-stimulated MDDC induced HIV-1-specific CD8 responses and protection from a virus challenge [34], [84]. On the other hand, it should be noted that the polypeptide Gag-Pol-Nef expressed by MVA-B-infected MDDC would have the recommended properties to induce strong HIV-specific immune responses in comparison with shorter and more unstable antigens, since long and stable mature protein antigens have been shown to be the best substrates for cross-presentation [78]. In view of that, in recent studies, it has been shown that infection of mice with MVA-B and the poxvirus vector NYVAC-B-C7L expressing the same HIV genes than MVA-B also induced mainly CD8^+^ T cell responses *in vivo*, thus highlighting the potential of this type of vectors to control latent and reactivating viruses [19], [85], [86].

In conclusion, we have shown that, upon MVA-B infection, MDDC can induce apoptosis, production of cytokines, antigen capture and maturation. MVA-B-exposed MDDC would migrate to lymph nodes and induce a highly functional HIV-specific CD8^+^ T cells response capable to effectively eliminate HIV-infected cells. Therefore, these results support the clinical research of prophylactic and therapeutic MDDC and MVA vaccines and, in particular, of MVA-B based vaccines against HIV infection.
